# Idiopathic facial lipoatrophy in a healthy middle-aged woman: a case report

**DOI:** 10.1186/s40463-019-0382-3

**Published:** 2019-11-14

**Authors:** Chad Purcell, S. Mark Taylor

**Affiliations:** Division of Otolaryngology - Head & Neck Surgery, Dalhousie University, Halifax, NS Canada

**Keywords:** Facial plastic surgery, Lipodystrophy

## Abstract

**Background:**

Facial lipoatrophy is a rare condition described by the disappearance of facial subcutaneous fat. The etiology of lipoatrophy can be congenital, or acquired including traumatic, iatrogenic or idiopathic. Idiopathic facial lipoatrophy has only been previously identified in three case reports, among which, the patient demographics vary considerably. Two of these case reports have identified a role for autologous fat transfer as a means of treatment. This case differs from those in the literature in patient demographics and severity of the facial lipoatrophy. The aim of the current report is to present a rare case of idiopathic facial lipoatrophy, and to assess the use of autologous fat transfer as a treatment modality.

**Case presentation:**

We present a case of a 40-year old woman from Nova Scotia, Canada who presented with asymptomatic idiopathic facial lipoatrophy. The patient was otherwise healthy, taking no medications with no trauma or surgery in the region affected. Investigations, including a full autoimmune workup, were unremarkable. The facial lipoatrophy was considerably disfiguring and was assessed as a Grade 4 on the facial lipoatrophy scale. The patient was treated over the course of 2 years with two autologous fat transfers.

**Conclusions:**

Achieving resolution of idiopathic lipoatrophy is important to patients because it can manifest in a disfiguring form and have negative effects on quality of life. The current study reports a treatment of idiopathic lipoatrophy that achieved results acceptable to the patient.

## Background

Lipodystrophy broadly refers to changes in the production, utilization, and storage of fat, and typically describes lipoatrophy or hyperadiposity. It is a defined by the disappearance of subcutaneous fat [1]. Lipodystrophy is broadly categorized into acquired and inherited forms. Acquired types are described as HIV-associated, partial generalized or localized lipodystrophy. Inherited types include congenital generalized lipodystrophy types 1 and 2, familial partial lipodystrophy in the Dunnigan variety or from PPARγ mutations, or type A or B mandibuloacral dysplasia. A detailed review of lipodystrophy and associated diseases can be found elsewhere [2].

Lipoatrophy is defined as disappearance of the subcutaneous fat, without exudative reactions or appreciable fibrosis and just as lipodystophy, it is divided into generalized, partial and localized forms [1]. Facial lipoatrophy describes the flattening or indentation of normally convex contours of the face [3] and the most commonly affected areas are the cheeks, temples and the preauricular, orbital or perioral region [4] It is associated with inheritable diseases, acquired diseases and the natural aging process [3, 4]. Congenital causes often involve a general absence of fat, with associated metabolic findings such as insulin resistance and acanthosis nigricans before adolescence [3]. The etiology of facial lipoatrophy is most commonly autoimmune in nature, but traumatic causes have also been identified [5–7]. Facial lipoatrophy has been described as a feature of other conditions. The most common conditions associated with facial lipoatrophy are antiretroviral therapy for HIV infection and connective tissue disorders that are associated with panniculitis such as lupus lupus erythematosus profundus and localized scleroderma also known as morphea [4]. Iatrogenic lipoatrophy is typically caused by medication injections. Further details on these other forms of lipodystrophy can be found elsewhere [3]. Idiopathic Facial lipoatrophy is a rare condition that results in aesthetic disfiguration. The stigmata and psychological effect this can have on patients has significant potential implications on a patients’ quality of life [3].

The severity of lipoatrophy ranges from mild facial flattening associated with aging, to severe facial depressions presenting as a manifestation of lipodystrophy from highly active antiretroviral therapy (HAART)-treated human immunodeficiency virus (HIV) patients [3]. The differences among etiologies of facial lipoatrophy presents a challenge in defining this presentation. To our knowledge there have been three previously reported cases of idiopathic facial lipoatrophy [1, 8, 9], two of which were treated with autologous fat transfer [8, 9]. The aim of the current report is to present a rare case of idiopathic facial lipoatrophy, and to assess the use of autologous fat transfer as a treatment modality.

## Case presentation

### Medical history

A 40-year-old woman was referred to the facial plastic surgery clinic with a seven-month history of asymptomatic left-sided facial lipoatrophy. She was otherwise healthy with no significant medical or surgical history and was not taking any medications. The lipoatrophy presented over the course of 2 weeks and remained stable for 6 months preceding presentation. There was no history of trauma, infection or previous surgery in the area. She did not receive treatment prior to presentation to clinic. The patient reported no previous lipoatrophy, HIV infection, or autoimmune disease. Her family history was unremarkable for these as well.

### Signs, symptoms and examination

**Fig. 1:**
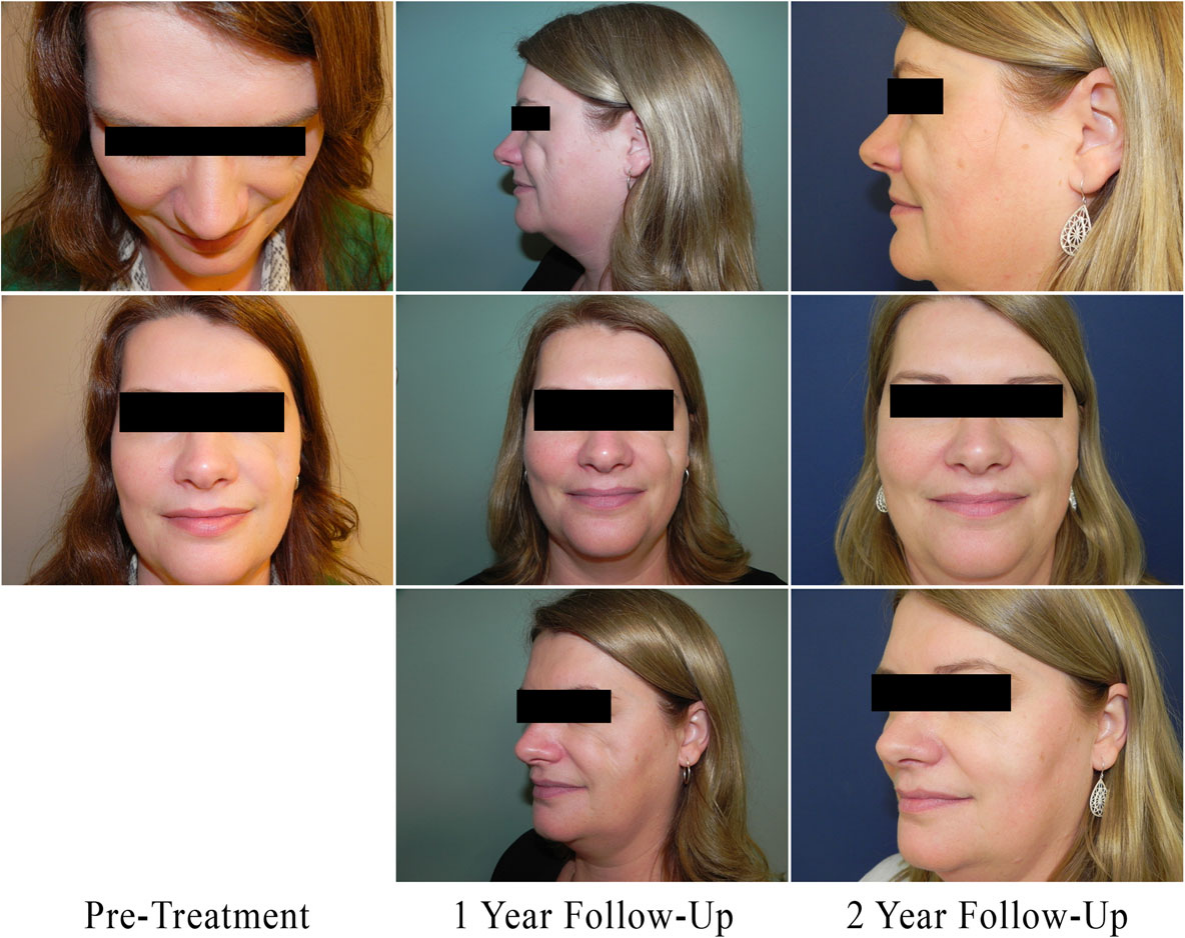
Treatment results indicating a sustained fullness of the left cheek after two treatments

The area of fat loss was asymptomatic but socially disfiguring (Fig. 1). On examination, there was almost complete loss of the malar fat pad over the malar eminence as well as a concave deformity extending temporally towards the lateral canthus. She was clinically assessed to have moderate to severe concavity of one or more facial regions, observable prominence of bony landmarks, and possible visibility of underlying musculature. These features were consistent with grade 4 facial lipoatrophy according to the scale developed by the Facial Lipoatrophy Panel [3]. Investigations, including a full autoimmune workup, were unremarkable. Lipoatrophy of this severity would be unlikely to resolve spontaneously. No other specialists were consulted in the investigation of this presentation.

### Treatment

The patient provided consent to treatment and the publication of this case report. She received two treatments of autologous fat grafting both of which were done using a Coleman type approach [10]. The area to be treated was marked with the patient standing. For the harvest of fat, 20 mL of 1% lidocaine with adrenaline was injected into the periumbilical area followed by initiation of sterile technique. Using a Coleman liposuction harvesting cannulae, a total of 15 mL of fat was harvested in an atraumatic fashion through a small periumbilical stab incision. The fat was not centrifuged, but was simply transferred directly into 1 mL leur lock syringes for injection, discarding any fluid that accompanied the fat. An infraorbital nerve block was performed with local anesthetic injection into the depressed area. The face was prepped and draped in a sterile fashion and a small stab incision was performed in the mid cheek subunit at the intersection of a vertical line drawn down from the lateral canthus and a horizontal line drawn from the ipsilateral alae. A total of 8 mL of fat was injected into the soft tissues of the cheek and various levels ranging from the supraperiosteum to the subcutaneous plane. Both umbilical and facial stab incisions were closed with a single fast absorbing gut suture. This procedure was repeated 1 year later as a second stage of treatment to improve the correction of the facial deformity. The patient reported no adverse effects from treatment and was satisfied with the results (Fig. 1). She continues to be followed annually though future treatments are not anticipated.

## Discussion and conclusions

Facial lipoatrophy describes the disappearance of facial adipose tissue. The appearance of this tissue loss can be socially and psychologically consequential, and often leads to patients seeking correction. The etiology of facial lipoatrophy can be genetic, acquired, natural aging or idiopathic. In this case, we describe an otherwise healthy 40-year-old woman presenting with severe acute-onset idiopathic hemi-facial lipoatrophy. The history, physical examination and autoimmune workup findings in this case did not warrant consultation of other services. However, we recommend approaching future cases on an individual basis and consider further medical workup as required prior to surgical intervention.

Traditional treatments of facial lipoatrophy included topical and oral medications for HAART-associated facial lipoatrophy, filler injections of hyaluronic acid, calcium hydroxyapatite, poly-L-lactic acid, or collagen. Autologous fat transfer appears to be an ideal alternative because it is biocompatibile with facial fat, procedurally simple to perform, does not cause allergic reactions, and can be done as an outpatient with a relatively short recovery [4]. However, drawbacks of autologous fat grafting include unpredictability of results due to the possibility of partial reabsorption of the fat graft over time [4]. Multiple treatments can help maintain the desired effect. In this case, the patient was re-treated with an additional grafting procedure 1 year after the initial treatment. Previous reports of autologous transfer of fat for idiopathic hemi-facial lipoatrophy have achieved similarly positive results [8, 9]. However, the patient characteristics vary considerably among these few studies.

This case features the detection and proposed treatment of a rare condition. To date, only three cases of idiopathic facial lipoatrophy have been reported [1, 8, 9]. A case report and literature review is the only possible methodology that can be used to report such rare presentations. Unfortunately, only poor-quality evidence exists for idiopathic facial lipoatrophy because the literature is comprised of only case reports and reviews. With such low numbers of known cases, high quality studies are all but precluded. Clinicians should consider lipoatrophy within their differential diagnoses to increase the number of known cases and help characterize this presentation. Larger studies may 1 day become more feasible and yield more case reports if we practice excellent detection and reporting of idiopathic facial lipoatrophy. The present case is supported by the literature that has reported good results when treating idiopathic facial lipoatrophy with autologous fat transfer [8, 9]. This case report marks a significant contribution to the lipoatrophy literature. Such contributions will improve understanding of lipoatrophy and the potential management options, as well as to stimulate. These results may be particularly of interest to facial plastic surgeons, who are positioned to be well equipped to manage this rare presentation. Much is still unknown about idiopathic facial lipoatrophy and future cases should be documented in the literature as a means of further characterizing this disease process.

## Data Availability

Data sharing not applicable to this article as no datasets were generated or analysed during the current study.
